# AITP-YOLO: improved tomato ripeness detection model based on multiple strategies

**DOI:** 10.3389/fpls.2025.1596739

**Published:** 2025-05-26

**Authors:** Wenyuan Huang, Yiran Liao, Peiling Wang, Ziao Chen, Ziqi Yang, Lijia Xu, Jiong Mu

**Affiliations:** ^1^ College of Information Engineering, Sichuan Agricultural University, Yaan, China; ^2^ Sichuan Key Laboratory of Agricultural Information Engineering, Yaan, China; ^3^ College of Mechanical and Electrical Engineering, Sichuan Agricultural University, Yaan, China; ^4^ Country College of Law, Sichuan Agricultural University, Yaan, China

**Keywords:** target detection, image recognition, YOLOv10, small target detection head, multi-scale, tomato

## Abstract

**Introduction:**

This paper offers a multi-scale AITP-YOLO model, based on the enhanced YOLOv10s model, to address the challenges of difficult identification and frequent misdetection of tomatoes, facilitating ripeness detection under realistic conditions.

**Methods:**

A four-head detector incorporates a small target detection layer, enhancing the model's capacity to identify small targets. Secondly, a multi-scale feature fusion technique employing cross-level features is implemented in the feature fusion layer to amalgamate convolutions of varying sizes, enhancing the model's fusion capacity and generalization proficiency for features of diverse scales. The bounding box loss function is modified to Shape-IoU, with the loss computed by emphasizing the shape and scale of the bounding box, hence enhancing the precision of bounding box regression, expediting model convergence, and augmenting model correctness. Ultimately, the model is compressed via Network Slimming puring,which removes redundant channels while mataining detection accuracy.

**Results:**

The experimental findings indicate that the enhanced model achieves average precision, accuracy, and recall of 92.6%, 89.7%, and 87.4%, respectively. In comparison to the baseline network YOLOv10s, the model weights are compressed by 7.64%, while average precision, accuracy, and recall are elevated by 4.6%, 5.8%, and 7.3%, respectively.

**Discussion:**

The enhanced model features a reduced model size while exhibiting superior detection capabilities, enabling more efficient and precise recognition of tomato stages amidst complicated backgrounds, hence offering a valuable technical reference for automated tomato harvesting technology.

## 1 Introduction

The precise identification of tomato ripening, as a significant economic crop, pertains not only to the market value of agricultural products but also to the safety and health of customers’ diets. China, being a leading global producer of tomatoes (Li et al., 2021), contributes 30% to the total worldwide output. Conventional tomato harvesting predominantly depends on manual expertise to assess ripeness, leading to diminished efficiency; this method is highly subjective, making it challenging to guarantee consistency and precision. With the expanding scale of tomato cultivation, which can span hundreds or even thousands of acres, relying on manual detection becomes inefficient, costly, and time-consuming. As agricultural technology progresses, the adoption of picking robots (Xiao et al., 2024) is increasingly replacing manual harvesting. Given the brief harvesting period of tomatoes, challenges in storage, and the potential health risks posed by residual pesticides (El-Sheikh et al., 2023), the robot must possess the capability to accurately classify tomatoes based on their varying stages of ripeness to facilitate timely harvesting, thereby significantly improving farmers’ income. Consequently, enhancing model detection efficiency is crucial for achieving automated tomato harvesting.

Recent technological advancements have generated much research on automated systems for agricultural chores, frequently employing image processing for the assessment of tomato quality (Gastélum-Barrios et al., 2011), with a particular emphasis on tomato ripeness detection. Conventional computer vision detection algorithms assess tomato maturity by analyzing fruit color (Fawzia Rahim and Mineno, 2021), size (Nassiri et al., 2016), and firmness (Alenazi et al., 2020) in images. The detection process is hindered by inadequate adaptability to complex environments, insufficient model generalization, and constraints in feature extraction, resulting in significant restrictions in detection speed and recognition accuracy, which fail to satisfy practical requirements.

As people’s needs continue to evolve, deep learning techniques are progressively transitioning toward smart agriculture technologies, with rising applications in fruit recognition, hence offering enhanced possibilities for tomato maturity detection. Vasconez et al. (2020) introduced Faster R-CNN with Inception V2, attaining a fruit counting performance of up to 93%, and SSDs combined with MobileNet, obtaining a performance of up to 90.0%, hence enhancing decision-making in agricultural practices. Jiang et al. (2021) employed a semi-supervised hyperspectral imaging technique to differentiate tomato ripeness, achieving a discrimination accuracy of 96.78%. Wang et al. (2022) enhanced the Faster R-CNN model with MatDet to augment recognition accuracy in intricate situations by addressing the issue of imprecise bounding box localization. Despite demonstrating high detection accuracy and minimal leakage rates, these approaches entail greater complexity in processing and reduced detection speed, hence hindering their capacity to fulfill real-time processing needs.

With ongoing advancements in machine vision, YOLO (You Only Look Once) demonstrates significant potential in agriculture due to its superior real-time performance, effective use of global information, balanced high accuracy and efficiency, and adaptability for real-time target detection in complex environments. Liu et al. (2020) employed a circular bounding box (C-Bbox) for tomato localization in their study on tomato ripeness identification and classification, as opposed to the conventional rectangular bounding box. They developed a robust detection algorithm based on YOLOv3, achieving a correct identification rate of 94.58% under conditions of slight occlusion. Nonetheless, the model possesses significant capacity, complicating deployment. Zeng et al. (2023) developed a lightweight tomato target detection algorithm and installed an Android-based real-time monitoring application for tomatoes, facilitating deployment in practical circumstances and enabling real-time observation. Kim et al. (2022) created an autonomous robotic system for tomato harvesting that incorporates a three-tier ripeness classification and 6D bit position estimation for the target fruits, achieving a collection success rate of 84.5%, thereby addressing issues related to large model size and the challenges of implementing practical robotic vision systems. Appe et al. (2023) devised a tomato detection model utilizing YOLOv5, which incorporated the CBAM attention mechanism into the network architecture and employed DIoU with NMS to mitigate the leakage rate of overlapping tomatoes. The proposed algorithm achieved an accuracy rate of 88.1%; however, this study also encounters the issue of low detection accuracy. Chen et al. (2024) introduced a multi-task deep convolutional neural network for detecting the maturity of cherry tomato bunches, incorporating two supplementary decoders into YOLOv7 for fruit ripeness identification, achieving a recognition accuracy of 86.6%. Wang et al. (2024b) introduced an enhanced YOLOv8s network for the real-time detection and segmentation of tomato fruits at various ripening stages, incorporating a variable focus loss (VFL) function alongside the regression loss function of Wise-IoU to address detection challenges, achieving an average precision of 91.4% (mAP@0.5). The processing velocity for simultaneous detection and segmentation was 60.2 frames per second. (Li et al., 2024) proposed a lightweight detection framework D^3^-YOLOv10 based on YOLOv10, in which multiple adaptive convolutional kernels are aggregated to extract locally effective features to adapt to the fruit size, which more effectively meets the needs of real-time tomato detection with a detection speed of 80.1 FPS.

The aforementioned study demonstrates the viability of recognizing and detecting tomato ripening with deep learning; nonetheless, some issues remain: Various sizes, both proximal and distal, together with diminutive targets and intricate shapes, are frequently misdiagnosed, excluded, and susceptible to inaccurate detection; the model architecture is intricate and comprises a substantial number of factors; furthermore, the recognition accuracy diminishes in complex scenarios. This research presents a novel AITP-YOLO model to address the aforementioned issues. The model may achieve excellent detection accuracy while being lightweight. This manuscript presents the subsequent key contributions:

1. A tomato ripening detection model, termed AITP-YOLO, was suggested, distinguished by its high accuracy and lightweight construction in complex backgrounds.
2. The incorporation of a diminutive target detection head enhances the model’s capacity to identify minute targets; employing a multi-scale feature fusion strategy amalgamates convolutions of varying dimensions, thereby augmenting the model’s generalization capability; modifying the bounding box loss function to Shape-IoU, which assesses loss by emphasizing the shape and scale of the bounding box, results in more precise bounding box regression.
3. The pruning technique known as Network Slimming eliminates “unimportant” channels by sparsifying the scale factor, with the objective of compressing the model, enhancing accuracy, and further diminishing model complexity.

## 2 Materials and methods

### 2.1 Data set

#### 2.1.1 Data acquisition

**Figure 1:**
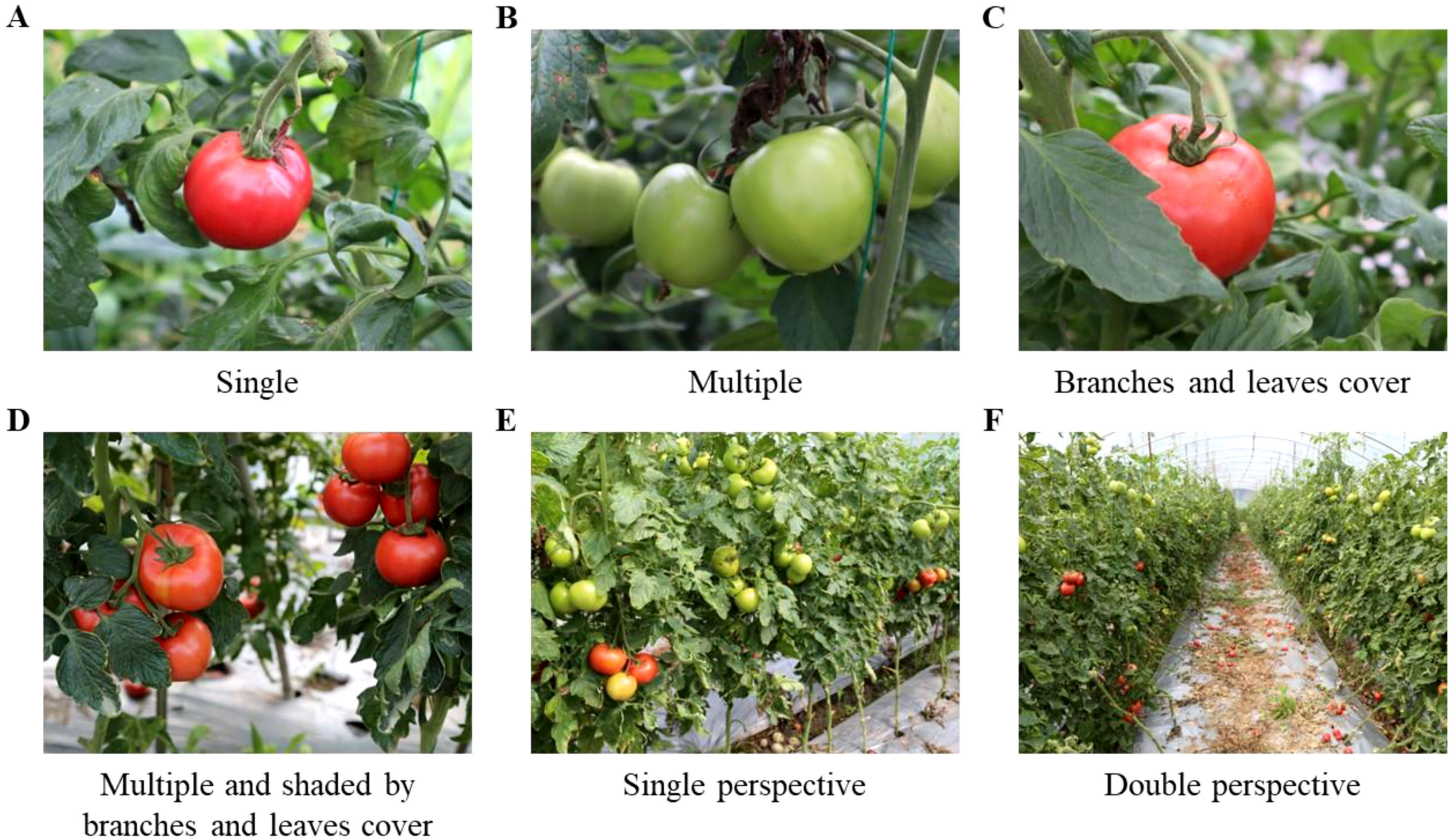
Dataset image type. **(A)** Single, **(B)** Multiple, **(C)** Branches and leaves cover, **(D)** Multiple and shaded by branches leaves cover, **(E)** Single perspective, **(F)** Double perspective.

The dataset was gathered from the Tomato Farm Picking Garden, Longhui Town, Neijiang City, Sichuan Province, China (102.99°N, 29.97°E). The shooting occurred between 2:00 p.m. and 4:00 p.m. in a greenhouse, ensuring consistent lighting conditions. A total of 3,154 pairs of images of tomatoes, featuring various plants and ripeness levels, were captured using a Canon R5 camera. The various shooting distances and angles for the plants resulted in the acquisition of single-fruit photographs, multi-fruit images, images featuring branch and leaf shade, one-sided lateral telephoto images, and two-sided forward telephoto images. Following the screening process, 3107 pairs of high-resolution images (4032 pixels × 3024 pixels) were acquired. **Figure 1** illustrates the primary categories of images within the dataset.

#### 2.1.2 Tomato ripening classes

The ripeness of tomatoes significantly influences their transportation, processing techniques, and flavor, making accurate classification of tomato ripeness essential. The maturity of tomato fruit, by agronomic standards and harvest criteria, is categorized into four stages: green ripening, color turning, firm ripening, and complete ripening. These stages are designated as green, turning, lighted, and red, respectively, as detailed in **Table 1**.

**Table 1:** Description of tomato ripening classifications.

Rank	Ripeness	RGB images	Labels	Explicit description
1	Green period	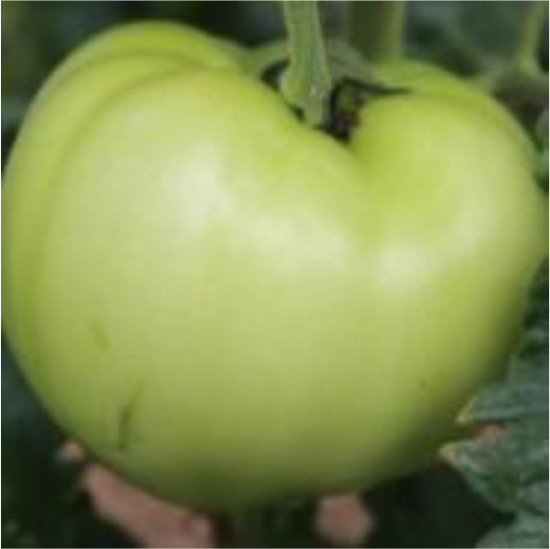	green	The fruit’s firm flesh and lime-green peel make it ideal for long-distance transportation.
2	Color change period	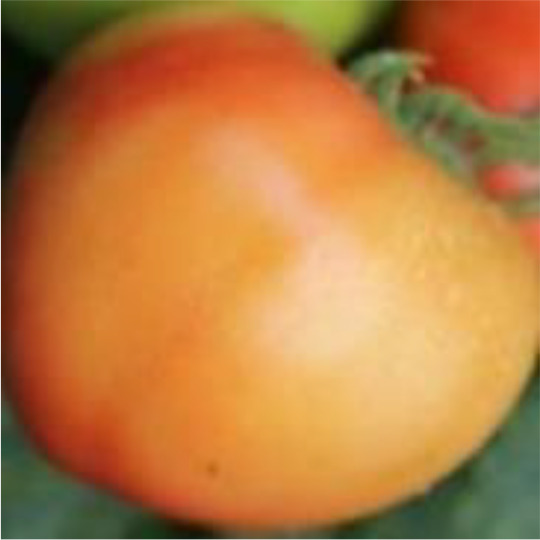	turning	The fruit becomes more suitable for short-distance transportation as its surface turns from green to light crimson and its sugar content rises.
3	Firm and ripe period	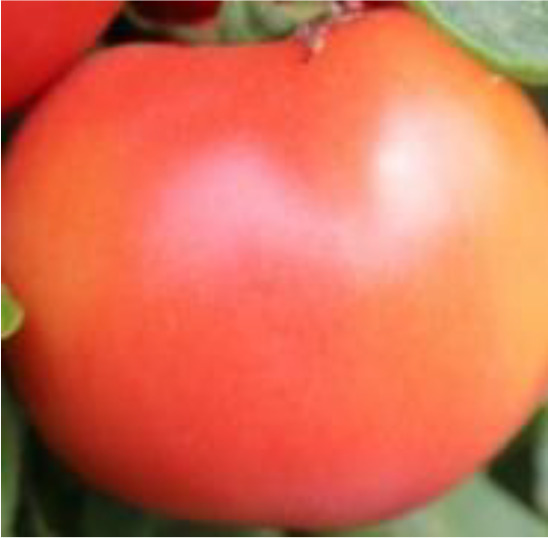	lighted	The fruit exhibits a red coloration over 75% of its surface area, indicating optimal ripeness for consumption and suitability for short-distance transport.
4	The fully ripe period	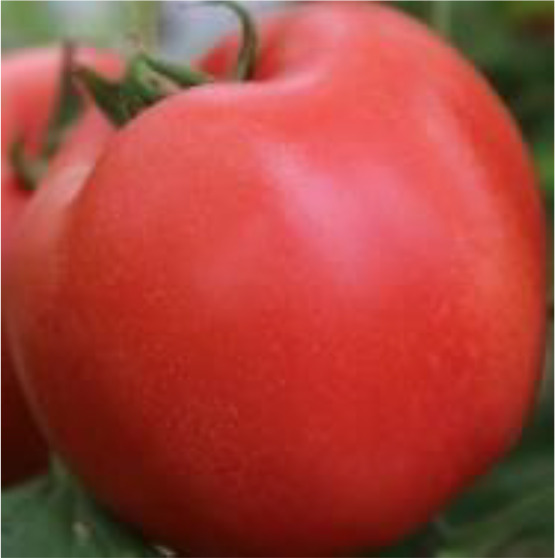	red	The fruit exhibits a uniform red coloration across its surface and possesses the highest sugar content, rendering it appropriate for proximity.

#### 2.1.3 Data preprocessing

**Figure 2:**
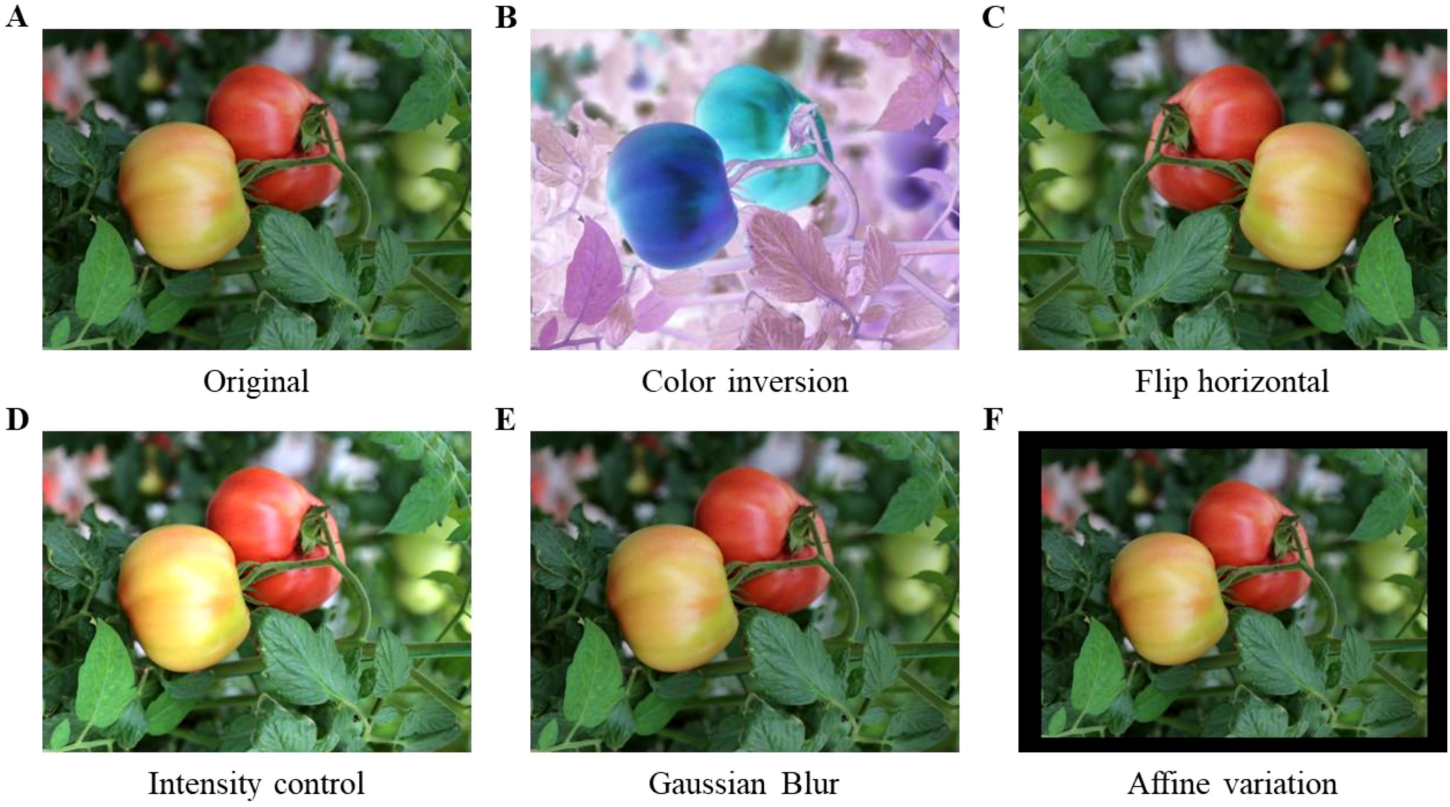
Effects of data augmentation. **(A)** Original, **(B)** Color inversion, **(C)** Flip horizontal, **(D)** Intensity control, **(E)** Caussian Blur, **(F)** Affine variation.

This study employs data augmentation techniques, including color inversion, level flipping, brightness adjustment, Gaussian blur, and affine transformation, to enhance network training efficacy, improve model generalization, and mitigate overfitting. The dataset is randomly combined and expanded, with several enhanced images depicted in **Figure 2**. Following data augmentation, a total of 6,214 sample images were acquired and randomly allocated into the training set and the test set in an 8:2 ratio.

The settings for the data augmentation approach are established as follows: Color inversion is accomplished by ‘IAA.Invert(0.3, per_channel=0.5)’, indicating a 30% application probability and a 50% independent inversion probability for each color channel (red, green, and blue), hence augmenting the model’s robustness to color information. The horizontal flip is executed using ‘IAA.Fliplr(0.5)’, with a 50% probability to simulate varying lateral observation effects, thereby augmenting data diversity. Brightness is adjusted to 1.3 times to replicate intensified lighting conditions, enhancing the model’s adaptability to diverse illumination environments. Gaussian blurring is applied with a standard deviation of 2.0 to mimic image distortion resulting from camera shake. The affine transformation translates the image by 15 pixels in both horizontal (x) and vertical (y) directions while scaling the image to 0.9 times its original size, which improves the model’s adaptability to target position variations and enhances generalization capability.

The datasets for this investigation were gathered in greenhouses in Southwest China under predominantly consistent light conditions. The primary attributes of tomatoes in this region include distinct color gradient variations throughout ripening, a regulated growth environment with natural shade, and variations in scale, rendering this datasets somewhat specific. To address the issue of geographic generalization, future studies may increase the datasets by gathering data from various regions or using transfer learning techniques to refine the model, enhancing its applicability across diverse geographic areas.

#### 2.1.4 Distribution of ripeness categories

Upon concluding data preparation, the picture data corresponding to the various ripening stages of tomatoes were meticulously quantified, with the count of image labels in each category and their respective percentages presented in **Table 2**. From the statistical results, it can be seen that the category distribution of the four stages of tomato ripeness (“green”, “turning”, “lighted” and “red”) is relatively balanced. The quantity of image labels in the green ripening stage is 15,487, representing 34.06%; in the turning stage, there are 8,909 labels, constituting 19.59%; in the firm ripening stage, the count is 9,047, accounting for 19.88%; and in the finished ripening stage, there are 12,023 labels, which comprise 26.48%. This more equitable distribution of categories can effectively mitigate the bias of a predominant category during model training, hence establishing a robust data foundation for steady training and precise detection in the succeeding model.

**Table 2:** Proportion of tomato ripeness labels.

Categories	Value	Ratio
green	15487	34.06%
turning	8909	19.59%
lighted	9047	19.88%
red	12023	26.48%

### 2.2 Experimental environment and parameter setting

The apparatus employed for the experimental training in this work consisted of two NVIDIA RTX 3080 GPUs. In the experiments, PyTorch 2.3.0, CUDA 12.1, and Python 3.8 were utilized, with the other hyperparameters configured as indicated in **Table 3**.

**Table 3:** Hyperparameter settings for the experiment.

Hyperparameter	Details
epochs	300
image size	640 × 640
batch size	16
workers	8
optimizer	SGD
lr0	0.01
lr1	0.01

### 2.3 Model evaluation metrics

In order to evaluate the performance and lightweight capability of the model, seven metrics were used in this study: precision (Equation 1), recall (Equation 2), mAP@0.5 (Equation 3), mAP@0.5:0.95 (Equation 4), GFLOPs (Equation 5), Weight, and FPS (Equation 6).


(1) $$\begin{array}{c} \text{Precision}=\frac{\text{True\_Positives }(\text{TP})}{\text{True\_Positives }(\text{TP})+\text{False\_Positives }(\text{FP})} \end{array}$$

(2) $$\begin{array}{c} \text{Recall}=\frac{\text{True\_Positives }(\text{TP})}{\text{True\_Positives }(\text{TP})+\text{False\_Negatives }(\text{FN})} \end{array}$$

(3) $$\begin{array}{c} \text{mAP}@0.5=\frac{1}{\text{N}}\sum_{\text{i}=1}^{\text{N}}\text{A}{\text{P}}_{\text{i}} \end{array}$$

(4) $$\begin{array}{c} \text{mAP}@0.5:0.95=\frac{1}{10}\sum_{\text{t}=0.5}^{0.95}m\text{AP}@\text{t} \end{array}$$

(5) $$\begin{array}{c} \text{FPS}=\frac{1}{\text{Inference\_Time\_per\_Frame}} \end{array}$$

Precision refers to the ratio of samples identified by the model as positive cases that are indeed positive, serving as a measure of prediction accuracy. It is calculated using True_Positives (TP) and False_Positives (FP) . Recall is the ratio of correctly predicted positive cases to the total actual positive cases, assessing the model’s effectiveness in identifying the positive class, and it accounts for False_Negatives (FN) . mAP@0.5 represents the average accuracy at an IoU threshold of 0.5, with higher values indicating superior model performance. Conversely, mAP@0.5:0.95 calculates the average accuracy across a range of IoU thresholds from 0.5 to 0.95 in increments of 0.05, providing a more comprehensive assessment of model performance under stringent accuracy conditions. GFLOPS quantifies the computational complexity of a model, indicating the number of floating-point operations executed per second, specifically in billions of floating-point operations. Weights denote the storage size of model parameters; a smaller size indicates a simpler model. FPS denotes the number of frames processed per second by the model or system, serving as a critical metric for assessing inference speed. Inference_Time_per_Frame indicates the duration necessary for the model to process an individual image frame.

## 3 Related work

### 3.1 Proposed AITP-YOLO object detection model

**Figure 3:**
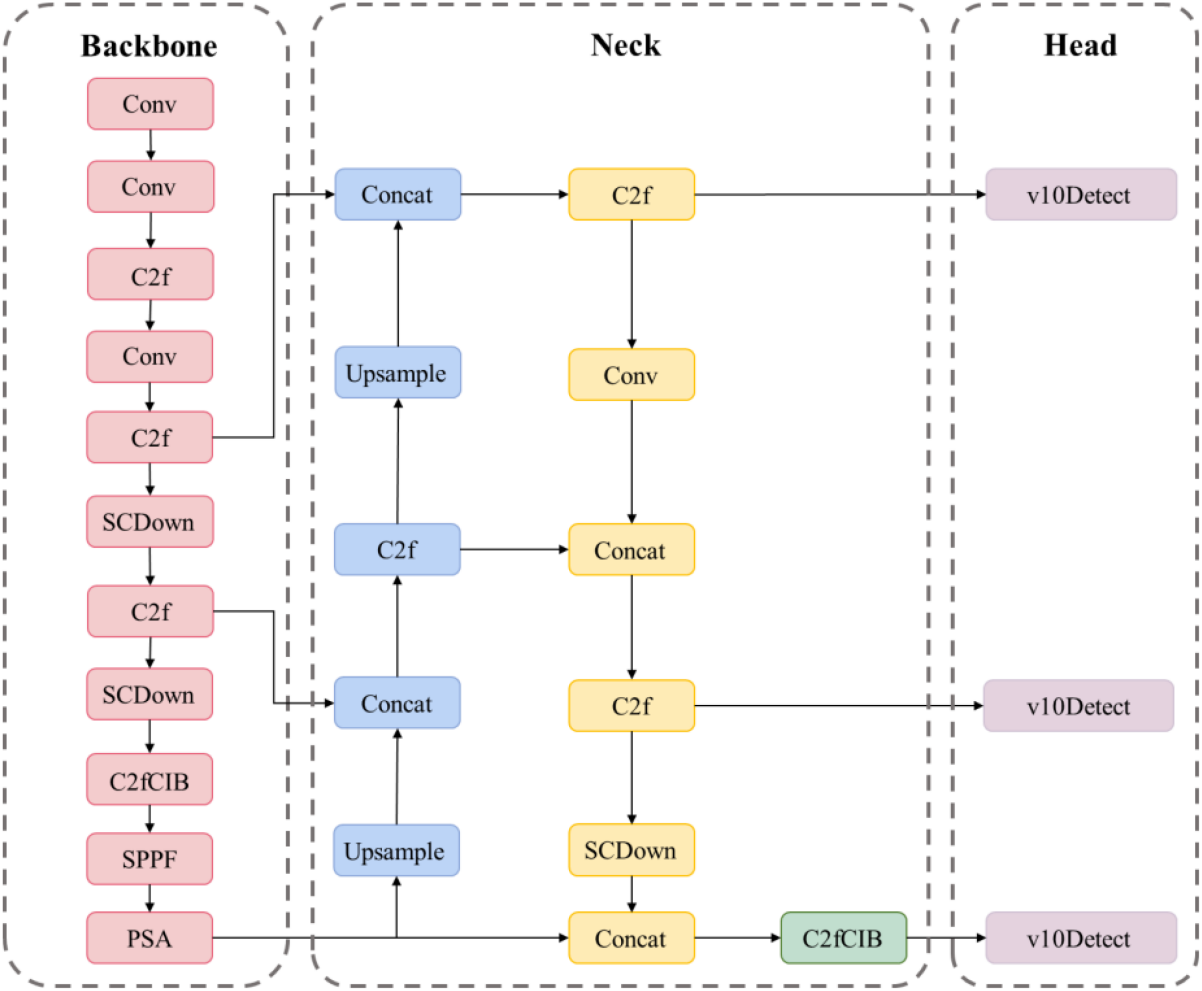
Structure of YOLOv10 network.

The YOLOv10 model (Wang et al., 2024a), depicted in **Figure 3**, is a real-time, end-to-end object detection model that markedly enhances accuracy and efficiency while preserving rapid detection through a novel dual-label assignment strategy and architectural advancements, thereby enabling real-time object detection and facilitating deployment and inference across various platforms. Nonetheless, precise detection of tomato fruits in intricate surroundings remains a significant challenge.

This study presents the AITP-YOLO model for tomato ripeness detection, employing multi-strategy enhancements to address the challenges of accurate identification and misdetection of tomatoes in natural conditions, thereby offering substantial support for automated tomato harvesting technology. The model is refined according to the YOLOv10s architecture, guaranteeing elevated accuracy alongside rapid detection speed.

**Figure 4:**
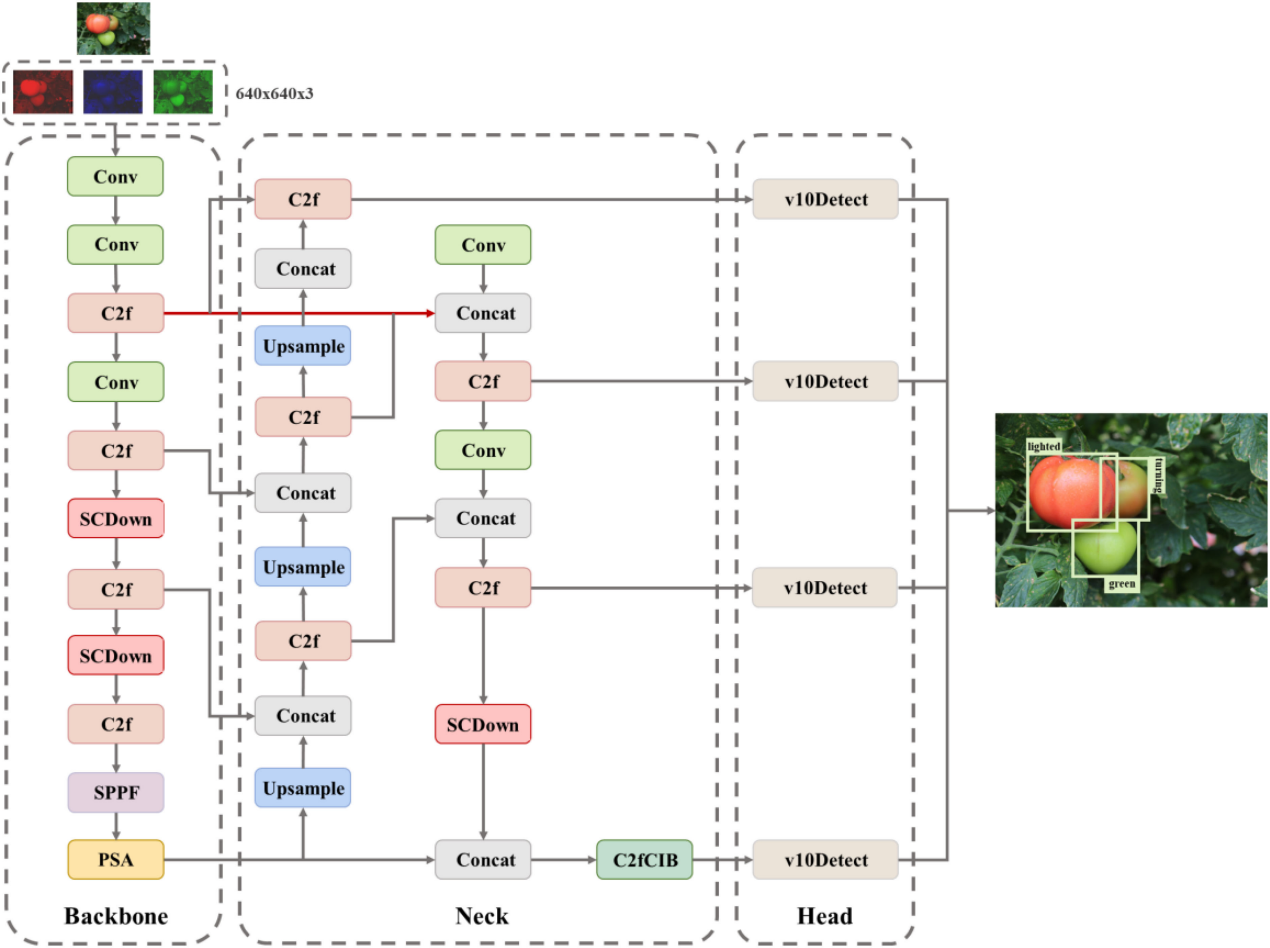
Structure of AITP-YOLO network.

The network architecture primarily consists of a trunk, neck, and head. The backbone component adopts the design of YOLOv10, so guaranteeing the model’s fundamental performance and stability, while effectively extracting essential characteristics from the image. The neck component employs a multi-scale strategy, integrating convolutions of varying dimensions to establish a bidirectional complementary connection between superficial detailed aspects and profound semantic information, thereby enhancing the capacity to represent multi-scale features. The head employs a four-head detection architecture and incorporates a P2/4-tiny detecting layer specifically designed for small targets. This design significantly improves the network’s capacity to identify minute targets. By integrating shallow and deep feature information, the model enhances its ability to accurately capture the intricate contour aspects of the target, hence improving recognition of small targets while preserving detection performance for medium- and large-sized targets. The model enhances the bounding box loss function by utilizing the Shape-IoU loss function in place of the original CIoU loss function. This approach emphasizes the shape and scale of the bounding box, resulting in more precise bounding box regression, expedited model convergence, and improved overall accuracy. The model is concurrently compressed utilizing the Network Slimming pruning technique, which diminishes model redundancy and complexity while enhancing detection accuracy. **Figure 4** depicts the architecture of the AITP-YOLO model developed in this research.

### 3.2 Four detection heads

This study’s tomato ripening datasets comprise a diverse array of image formats, featuring a substantial quantity of little target tomatoes. The abundance of small targets complicates the accurate identification of these targets by conventional models and their usual detection heads. The YOLOv10 model’s standard detection head comprises three layers: P3, P4, and P5, with each detector head tailored for targets of varying scales. The P3/8-small detector head possesses an extensive feature map and high resolution, primarily identifying small targets ranging from 8×8 to 16×16 pixels; the P4/16-medium detector head experiences greater downsampling and leverages an increased channel count to capture rich semantics, detecting medium targets from 16×16 to 32×32 pixels; the P5/32-large detector head exhibits the most robust semantic characterization and sensory field, identifying large targets of 64 pixels. The P5/32-large detector head possesses the most robust semantic characterization and sensory field, capable of detecting large targets of 64×64 pixels or greater.

**Figure 5:**
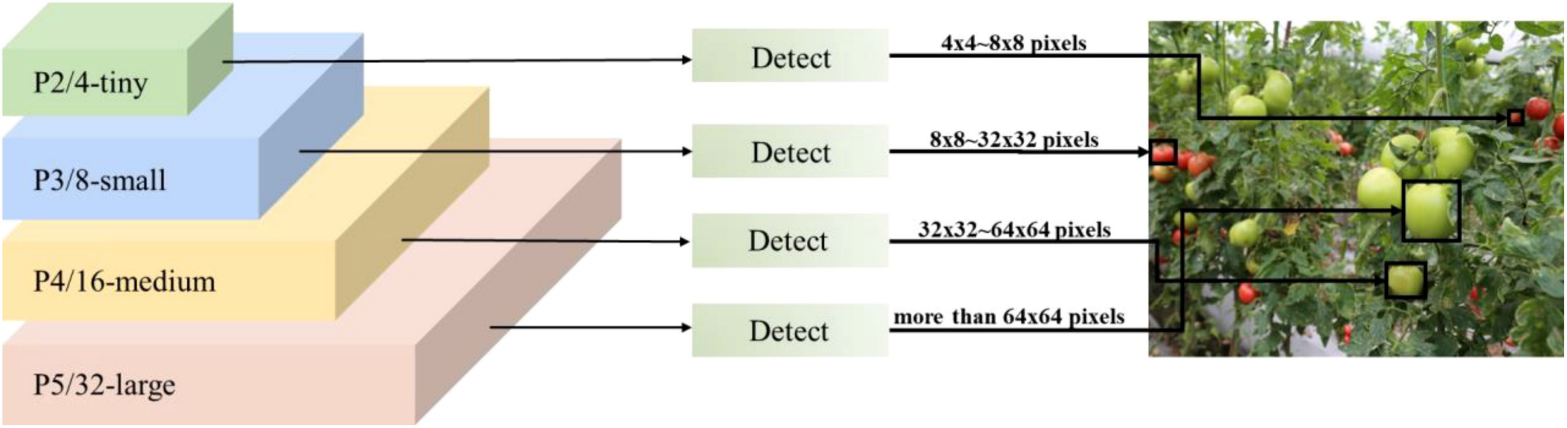
Schematic diagram of the four-head detection head.

Due to the large proportion of small targets in the datasets, to improve the accuracy of small target detection, a specialized small target detection head (Chen and Zhang, 2024) P2/4-tiny is introduced on top of the original three detection heads to form a four-head detection head architecture to better cope with the complexity of the datasets, which have a large number of small targets. This detection head is specifically optimized for tiny targets from 4×4 to 8×8 pixels, and this optimization effectively makes up for the shortcomings of the original model in tiny target detection. As shown in **Figure 5**, the four-head detection head significantly improves the model’s small-target detection capability by fusing shallow and deep feature information, especially enabling the model to better capture the detailed contour features of the target, thus improving the detection accuracy. This improvement not only enhances the model’s ability to recognize small targets but also maintains the performance advantage of the original detection head in medium and large target detection.

### 3.3 Multi-scale feature fusion

In natural settings, detecting tomato ripeness encounters numerous challenges: the visual characteristics of the turning and illuminated stages of tomatoes are similar, leading to potential confusion; accurately identifying fruit ripeness is complicated by overlapping fruits and obstructions from branches and leaves; additionally, there is considerable scale variability among tomato specimens, with pronounced size discrepancies across different growth stages and varieties, exacerbated by the effects of shadows and other environmental elements. Environmental factors, including shadow interference, considerably affect the accurate detection of tomato ripening.

This study addresses the scale diversity and environmental interference of tomato targets in complex natural scenes by proposing a multiscale feature fusion method for cross-level features (Quan et al., 2023). This method enhances the characterization capability of multiscale features by establishing a bidirectional complementary link between shallow detailed features and deep semantic information. The multi-scale feature fusion technique is employed at the feature fusion layer to integrate convolutions of varying dimensions. As indicated by the red (Xu, 2023) arrows in **Figure 1**, following extensive experimental modifications, the feature map output from the convolution operation of the twofold C2f module is concatenated and integrated with the feature map output from the convolution operation of the single C2f module. The resultant fused feature map maintains the convolutional scale while doubling the number of channels, thereby offering enhanced feature information for the subsequent target detection task.

During the feature extraction phase of the backbone network, low-level features encompass intricate details such as the texture and edge contours of the fruit’s surface, whereas deep-level features encapsulate high-level semantics, including the overall morphology of the fruit and the color distribution associated with ripeness. This fusion approach enables the efficient extraction of deep-level features to shallow-level features.

Multi-scale fusion is enhanced in three key dimensions:

1. By incorporating low-level spatial information, the model improves its ability to identify fine details, like the local color gradient of ripening fruits and contour remnants concealed by branches and foliage.;
2. Employ semantic guidance to leverage the ripening discriminative information in deep features to correct the tendency for misjudgment in shallow features under complex lighting circumstances.;
3. Dynamic adaptation: utilizing deformable convolution to establish the feature interaction layer, allowing the network to independently adjust the weights of the feature fusion regions to effectively tackle challenges such as fruit overlap and distant view deformation.

The experimental findings demonstrate that the multi-scale fusion method significantly improves the model’s mean average precision (mAP), especially in the small target detection task, where both recall and accuracy are notably increased. The multi-scale feature fusion technique, through multi-level feature interaction, enhances the model’s ability to understand targets at different scales, hence improving overall detection effectiveness. The model excels in target detection inside complex environments and dramatically improves the detection of small, unclear, and obscured targets. This technology is applicable not only for identifying tomato maturity but also provides substantial technological assistance for many other automated agricultural detection jobs.

### 3.4 Bounding frame loss function Shape-IoU

In the tomato target detection task, the dataset contains two types of images, near-view and far-view, where the tomato target in the far-view is smaller, and the loss function needs to be optimized to improve the model’s performance for detecting the ripeness of smaller tomatoes. The original YOLOv10s model uses the CIoU (Zheng et al., 2020) bounding box loss function, and CIoU is designed to take the shape information of the target box into account, but in the prediction box regression process, if the aspect ratio of the prediction box and the real box (Ground Truth box) is linearly proportional, the penalty in the CIoU loss function degrades to 0 and becomes ineffective.

The corresponding bounding box regression loss for CIoU (Equation 6) is calculated as follows:


(6) $$\begin{array}{c} {\text{L}}_{\text{CIoU}}=1-\text{IoU}+\frac{{\text{ρ}}^{2}\left(\text{b},\;{\text{b}}^{\text{gt}}\right)}{{\text{c}}^{2}}+\partial \text{v} \end{array}$$

where v is the parameter used to measure the consistency of the aspect ratio (Equation 7), defined as follows:


(7) $$\begin{array}{c} \text{v}=\frac{4}{{\text{π}}^{2}}{\left(\text{arctan}\frac{{\text{w}}^{\text{gt}}}{{\text{h}}^{\text{gt}}}-\text{arctan}\frac{\text{w}}{\text{h}}\right)}^{2} \end{array}$$

This study advocates for the utilization of the Shape-IoU (Zhang and Zhang, 2023) loss function in place of the CIoU loss function. Shape-IoU incorporates centroid distance and width-to-height ratio while also introducing shape similarity, a useful metric for assessing shape disparity by quantifying the variations in width and height between the anticipated and actual frames. Furthermore, the implementation of exponential decay processing allows the form loss to more accurately represent shape variations, mitigating the issue of the loss function being overly sensitive to minor discrepancies. This enhanced Shape-IoU loss function increases regression accuracy and accelerates model convergence, resulting in superior performance in complicated scenarios.

The corresponding bounding box regression loss for Shape-IoU (Equation 8) is calculated as follows:


(8) $$\begin{array}{c} {\text{L}}_{\text{Shape}-\text{IoU}}=1-\text{IoU}+\text{distanc}{\text{e}}^{\text{shape}}+0.5\times {\text{Ω}}^{\text{shape}} \end{array}$$

The distance shape calculation formula (Equation 9) is as follows:


(9) $$\begin{array}{c} \text{distanc}{\text{e}}^{\text{shape}}=\text{h}\text{h}\times {\left({\text{x}}_{\text{c}}-{\text{x}}_{\text{c}}^{\text{g}\text{t}}\right)}^{2}/{\text{c}}^{2}+\text{w}\text{w}\times {\left({\text{y}}_{\text{c}}-{\text{y}}_{\text{c}}^{\text{g}\text{t}}\right)}^{2}/{\text{c}}^{2} \end{array}$$

The shape loss calculation formula (Equation 10) is as follows:


(10) $$\begin{array}{c} {\text{Ω}}^{\text{shape}}=\sum_{\text{t}=\text{w},\text{h}}{\left(1-{\text{e}}^{-{\text{ω}}_{\text{t}}}\right)}^{\text{θ}},\text{θ}=4 \end{array}$$

**Figure 6:**
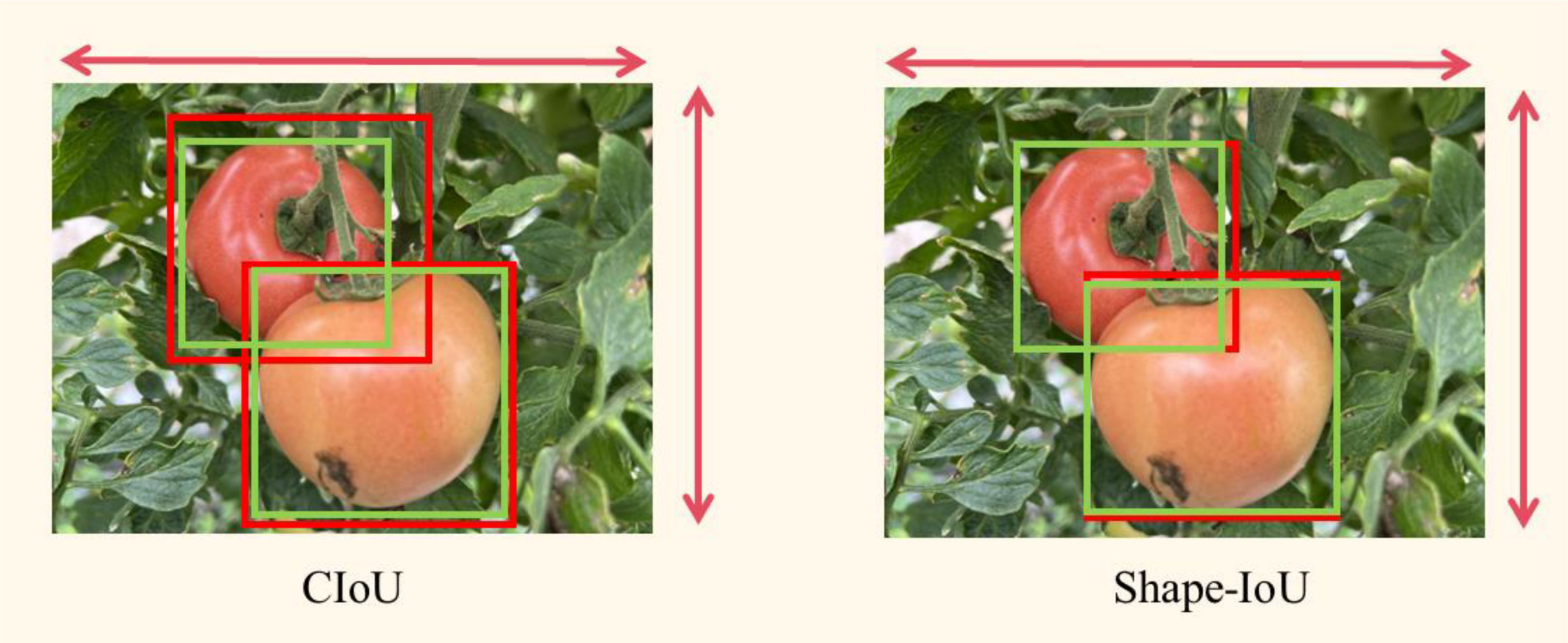
Comparison between the CIoU loss function and the Shape-IoU loss function.

**Figure 6** illustrates the efficacy of CIoU and Shape-IoU in practical scenarios, with red representing the actual bounding box and green denoting the anticipated bounding box. Although the CIoU loss function incorporates the aspect ratio to enhance bounding box shape alignment, it fails to ensure precise identification of targets with varying shapes. In contrast, Shape-IoU emphasizes both the shape and size characteristics of the bounding box, effectively capturing the intrinsic attributes of diverse targets (e.g., aspect ratio, area) by directly modeling the discrepancies in the geometric properties of predicted and actual boxes, thereby enhancing regression accuracy.

### 3.5 Prune

#### 3.5.1 The principle of pruning

The enhanced model for tomato ripeness detection markedly increases detection accuracy while maintaining minimal alterations in model size and computational demands; however, redundancy persists due to its backbone and convolutional architecture, resulting in a substantial resource burden on prospective embedded devices. To enhance the model’s efficiency and accelerate inference, the Network Slimming (Liu et al., 2017) approach is employed for model compression.

In the implementation of the Network Slimming approach, the Batch Normalization (BN) layer in the network model is first sparsely trained to eliminate insignificant channels, which are autonomously recognized and pruned during training to attain rapid convergence and enhanced generalization performance. The Network Slimming method introduces a scaling factors ( γ ) for each channel, which is multiplied with the output of the channel as a sparsity-induced penalty term, which is combined with the normal training loss, and then sub-gradient descent is used as an optimization tool to drive the model training process toward the desired goal of efficient evolution. The loss function L (Equation 11) is formulated as follows:


(11) $$\begin{array}{c} \text{L}=\sum_{\left(\text{x},\text{y}\right)}\text{l}\;\left(\text{f}\left(\text{x},\text{W}\right),\text{y}\right)+\text{λ}\sum_{\text{γ}\in \text{Γ}}g\left(\text{γ}\right) \end{array}$$

In this context, (x,y) represents the training input and goal, W signifies the trainable weights, g(γ) symbolizes the penalty function for the scaling factor, and λ indicates the equilibrium factor of the two components, namely the sparsity rate. In this study, g(s) is defined as |s| , corresponding to the L1 paradigm number.

#### 3.5.2 Model pruning steps

**Figure 7:**
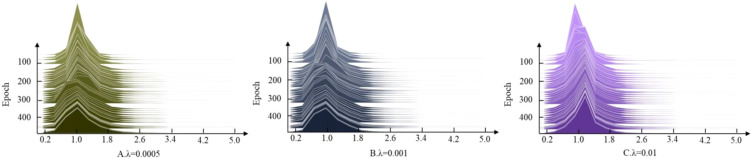
Setting the change in scaling factor γ for different regular term coefficients λ . **(A)** λ=0.0005 , **(B)** λ=0.001 , **(C)** λ=0.01 .

The hyperparameter values influence the extent of L1 regularization applied to the scaling factor of the batch normalization layer during training, thus affecting the weights of the model’s BN layer about the average accuracy. To ascertain the suitable scaling factor, λ , which governs the intensity of sparse regularization, values of 0.0005, 0.001, and 0.01 are assigned for the sparse experiments. The results, depicted in **Figure 7**, demonstrate that λ = 0.01 approaches 0, exhibiting the highest speed and optimal sparse effect. Because the tomato dataset has many categories, rich data, complicated structure and low redundancy, λ = 0.01 is finally adopted for the sparse regularization operation.

After the sparse training phase, channels with scaling factors below this threshold and their associated connections and weights are removed, resulting in a more compact model structure. Since the pruning process may trigger a decrease in model accuracy, it is necessary to ensure the model accuracy with the help of fine-tuning means. Meanwhile, through multiple iterations of pruning and training, after each pruning, the narrow network is re-trained with sparse regularization, and then the pruning operation is implemented again to gradually realize the deep compression of the model.

## 4 Results and analysis

### 4.1 Detection head comparison experiment

To further validate the impact of the four-head detection head on model performance, comparative experiments were conducted using two-head, three-head, and four-head detection heads using the dataset of this study. The P3/8-small detection head, utilized for identifying small targets, has been removed from the two-head detection head trials. The findings are presented in **Table 4**.

**Table 4:** Comparative experiment of three types of detection heads.

Detection head	P/%	R/%	mAP@0.5/%	mAP@0.5:0.95/%
two-detection heads	85.6	78.6	86.2	64.4
three-detection heads	83.9	80.1	88	69.1
four-detection heads	86.8	84.8	91	74

Analysis of the results in **Table 4** reveals that all indices of the dual-head detection system are inferior to those of the conventional triple-head detection system, hence underscoring the critical importance of small target identification in the experiment. In comparison to the regular three-head detection head, the four-head experimental head enhances accuracy, recall, mAP@0.5, and mAP@0.5:0.95 by 2.9%, 4.7%, 2%, and 4.9%, respectively. The model with an additional small target detection head demonstrates superior detection outcomes and markedly improves its capacity to identify minute targets.

### 4.2 IoU loss function comparison experiment

**Figure 8:**
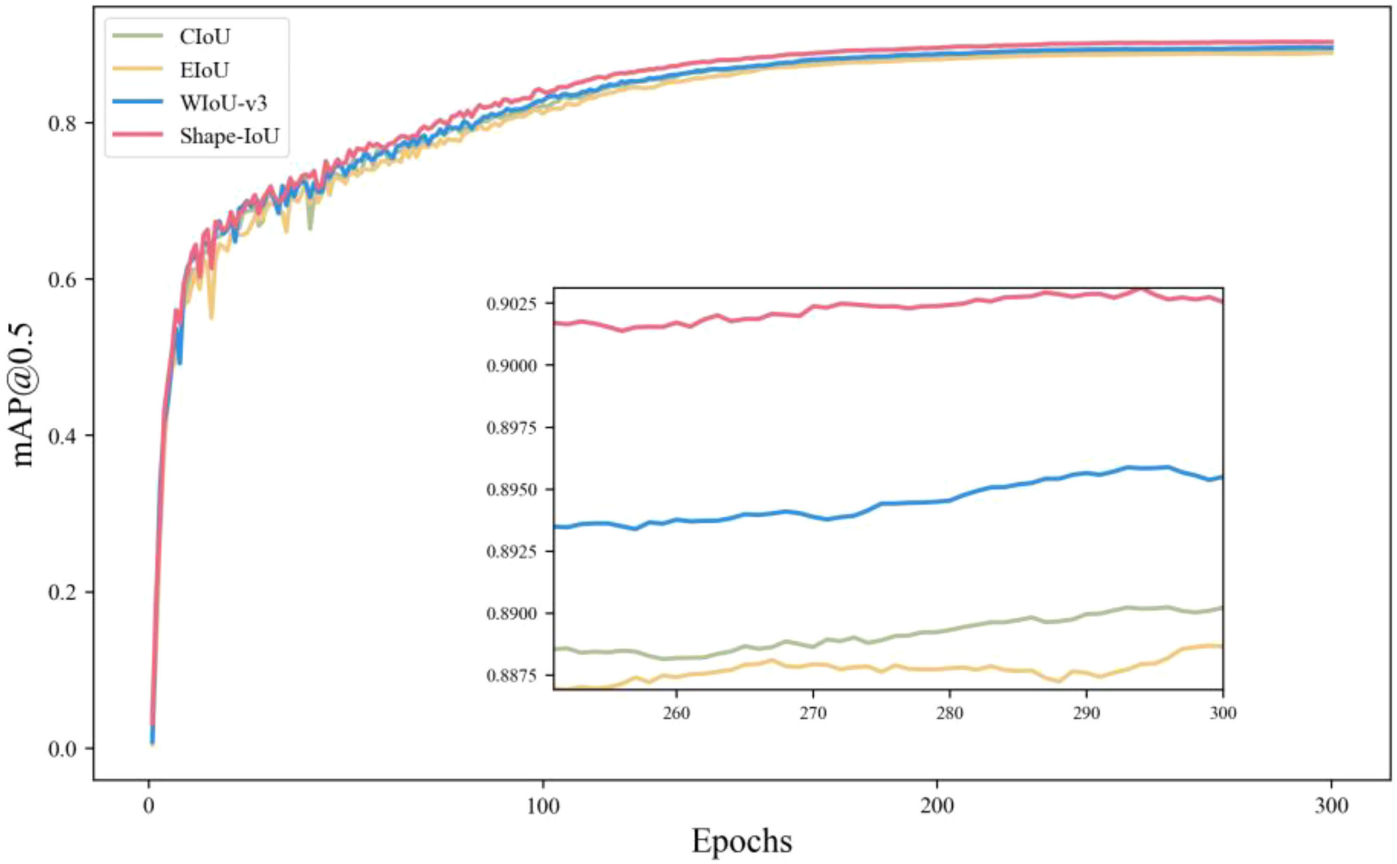
Comparison of loss functions.

To further validate the generalization and efficacy of the Shape-IoU loss function, YOLOv10s serves as the experimental benchmark model. The Shape-IoU loss function is evaluated against currently prevalent loss functions, including CIoU, EIoU (Zhang et al., 2022), and WIoU-v3 (Tong et al., 2023), utilizing the dataset of this study, with results illustrated in **Figure 8**.

**Figure 8** illustrates that the Shape-IoU loss function demonstrates a significant advantage in the mAP@0.5 metric without augmenting the model parameters or computational demands. This is attributable to its shape similarity metric mechanism, which enhances matching accuracy by precisely quantifying the shape disparity between the predicted and actual frames, thereby enabling Shape-IoU to excel in scenarios involving significant shape variations and to be more suitable for detection tasks involving small targets and extreme shapes. The experimental results indicate that the Shape-IoU loss function surpasses the prevailing mainstream loss function in the tomato dataset characterized by a complicated background and ambiguous maturity.

### 4.3 Comparison of pruning effects

This research compares the Network Slimming pruning approach with two other pruning techniques: LAMP (Lee et al., 2020) and Pruning Filters (Filters’importance, D, 2016). Eight distinct trimming rates of 5%, 10%, 15%, 20%, 25%, 30%, 40%, and 50% were established for the experiment, with the comparative results presented in **Table 5**. The experimental results derived from the two methodologies, LAMP and Pruning Filters, indicate that model compression and a corresponding enhancement in accuracy cannot be concurrently attained, even under optimal conditions for each method. Network Slimming has superior attributes; hence, this method is selected for executing pruning operations on the model in this study.

**Table 5:** Comparison of pruning results.

Pruning method	Pruning rate	P/%	R/%	mAP@0.5	mAP@0.5:0.95	Weight/MB	GFLOPs
None (v10s-AITP)	0	88.7	86.4	92.1	70	16.3	37.3
LAMP	50%	42.8	41.1	35.8	23.5	8.22	16.2
	40%	85.1	79.7	87.8	69	9.98	19.2
	30%	88.9	85	91	74.4	11.4	22.8
	25%	89.2	85.5	91.4	75.6	12.3	25.4
	20%	90.1	86.6	92.1	77	13.1	27.7
	15%	91	86.4	92.3	77.6	13.8	29.6
	10%	90.3	86.9	92.2	77.8	14.5	31.5
	5%	89.9	87.8	92.2	78.4	15.4	33.8
**Network Slimming**	50%	43.9	40.7	37.4	24.8	8.22	16.2
	40%	81.5	78.3	85.7	66.1	9.98	19.2
	30%	88	83.1	90	72.9	11.4	22.8
	25%	88.5	86.6	91.8	76.5	12.3	25.4
	20%	89.8	86	91.8	76.6	13.1	27.7
	15%	89.9	87.1	92.1	77.4	13.8	29.6
	**10%**	**89.7**	**87.4**	**92.6**	**78.2**	**14.5**	**31.5**
	5%	89.9	87.4	92.2	78.3	15.3	33.6
pruning filters	50%	43.9	41.5	37	24.7	8.22	16.2
	40%	84.4	78.6	86.6	67.3	9.98	19.2
	30%	88.8	84.2	90.8	73.8	11.4	22.8
	25%	89.3	85.2	91.3	75.3	12.3	25.4
	20%	90.6	84.9	91.9	76.5	13.1	27.7
	15%	90.3	86.1	91.9	77.4	13.8	29.6
	10%	90.5	86.8	92.3	78	14.5	31.5
	5%	90.4	871.	92.4	78.2	15.3	33.6

The bolded values indicate the best results obtained using the Network Slimming (10% pruning rate) pruning method.

In the experiments conducted on Network Slimming, it was observed that when the pruning rate is less than 10%, the network slimming retains most of the important channels, which does not have much impact on the accuracy. When the pruning rate is more than 10%, although the number of model parameters decreases more obviously, the loss of accuracy is correspondingly more, and when the pruning rate is too high, the accuracy also decreases but the number of parameters decreases less. Specifically, when the pruning rate surpasses 20%, the detection accuracy decreases linearly as the pruning rate increases, due to a greater number of channels in the detection layer for small targets and fewer channels in the effective layer, resulting in the cropping of effective channels. When the pruning rate surpasses 20%, detection accuracy declines linearly due to an increase in channels within the small target detection layer and a reduction in channels inside the effective layer. A pruning rate of 10% is ultimately used to optimize the equilibrium between model compression and accuracy.

**Figure 9:**
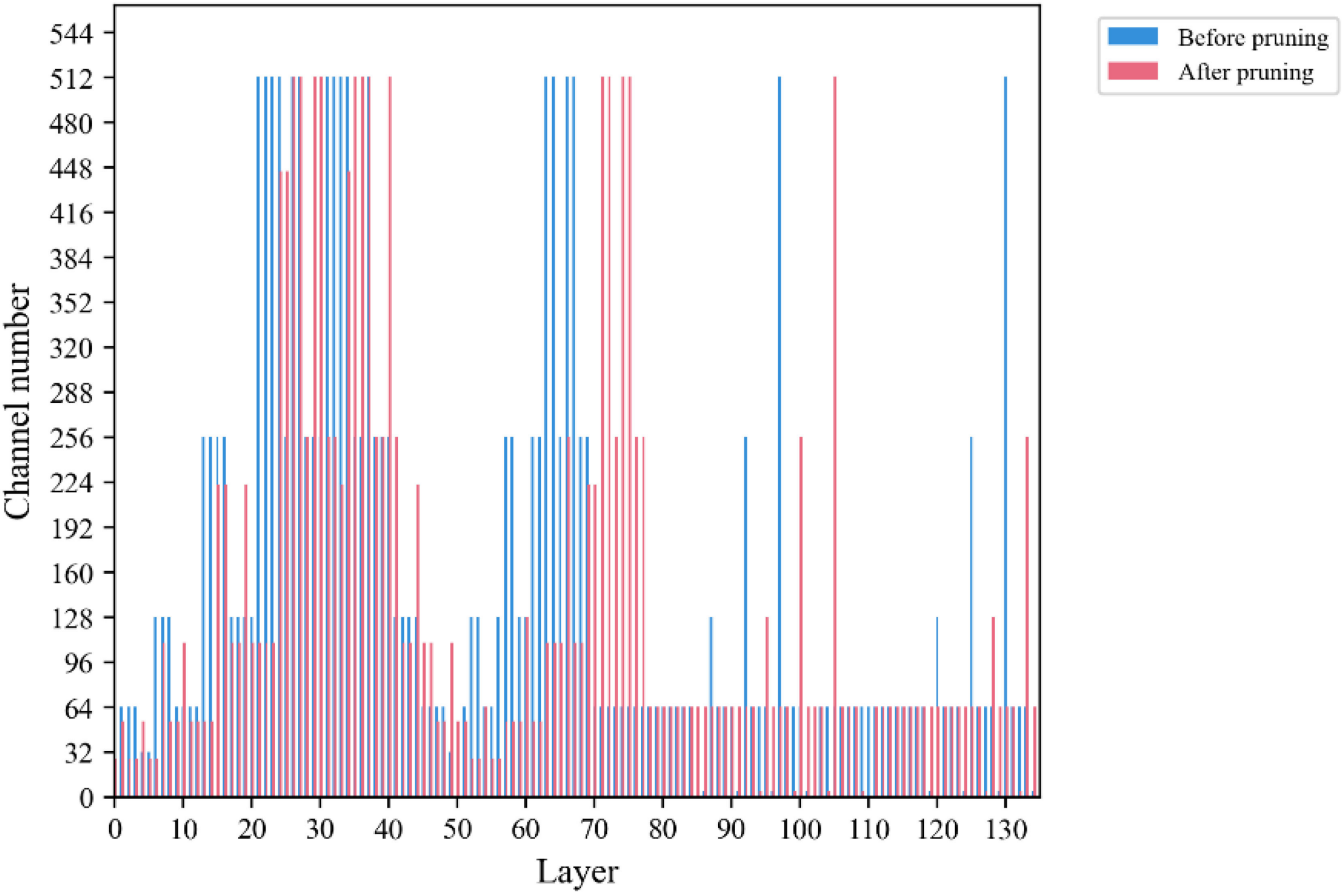
Comparison of channel number.

After pruning using the Network Slimming method, the number of channels in the front and back layers is shown in **Figure 9**, which shows that the number of channels in “Before pruning” is generally higher than that in “After pruning” in each layer, which indicates that the pruning operation has reduced the number of channels in the model to a certain extent. After the experiment, it can be found that most of the channels are compressed to a certain extent, and the results show that the pruning algorithm is effective for the model.

### 4.4 Ablation experiments

This work established a standardized environment and experimental conditions for eight sets of ablation experiments, with the objective of thoroughly and precisely evaluating the effects of various techniques on tomato ripening detection. The YOLOv10s model was chosen as the baseline and assessed using eight assessment measures, with the results presented in **Table 6**.

**Table 6:** Results of ablation studies for the improved model.

Numbers	Four-detection heads	Multi-scale	Shape-IoU	P/%	R/%	mAP@0.5/%	mAP@0.5:0.95/%	Weight/MB	GFLOPs	FPS
**1**	×	×	×	83.9	80.1	88	69.1	15.7	24.8	103.09
**2**	**√**	×	×	86.8	84.8	91	74	16.2	37.1	92.59
**3**	×	**√**	×	87.6	82.7	89.9	72.7	15.9	25	106.84
**4**	×	×	**√**	89	82.9	90.3	67.7	15.7	24.8	102.3
**5**	**√**	**√**	×	87.8	84.8	91.1	74.4	16.3	37.3	96.89
**6**	**√**	×	**√**	88.2	85.9	91.2	68.5	16.3	37.1	94.16
**7**	×	**√**	**√**	87.6	83.7	89.7	67.5	15.9	25	108.45
**8**	**√**	**√**	**√**	**88.7**	**86.4**	**92.1**	**70**	**16.3**	**37.3**	**95.97**

“×”This policy is not used; “√” to use this policy.The bold values indicate the optimal results in the ablation experiment.

From the results in **Table 6**, it can be seen that Experiment 1 uses the baseline model YOLOv10s, the precision of ripeness detection for multi-stage tomato is 83.9%, the recall is 80.1%, the mAP@0.5 is 88%, the mAP@0.5:0.95 is 69.1%, the model weight is 15.7MB, the floating-point operation is 24.8GFLOPs, and the FPS is 103.09; Experiment 2 uses the four head detection heads and adding small target detection heads, the precision, recall and mAP@0.5 are improved by 2.9, 4.7 and 3 percentage points respectively compared to Experiment 1, but the model weights are elevated; Experiment 3 uses a multi-scale fusion method, the mAP@0.5 is improved by 1.9 percentage points, which improves the detection precision by a small margin, and the frame rate is improved by 3.64%; Experiment 4 replaces the Shape-IoU loss function, the precision, recall and mAP@0.5 are improved, indicating that Shape-IoU helps to accelerate model convergence and improve model precision; experiment 8 using a combination of the three strategies achieves the best detection effect with the improved model compared to the baseline network, with the precision, recall, and mAP@0.5 improved by 4.8, 6.3, and 4.1 percentage points, respectively, and the detection performance is significantly improved, even though the model weight and the amount of operations are increased, improved significantly. The comprehensive ablation experiment results demonstrate the positive significance of the optimization strategy proposed in this study for the YOLOv10s network.

### 4.5 Comparison of model performance before and after improvements

**Figure 10:**
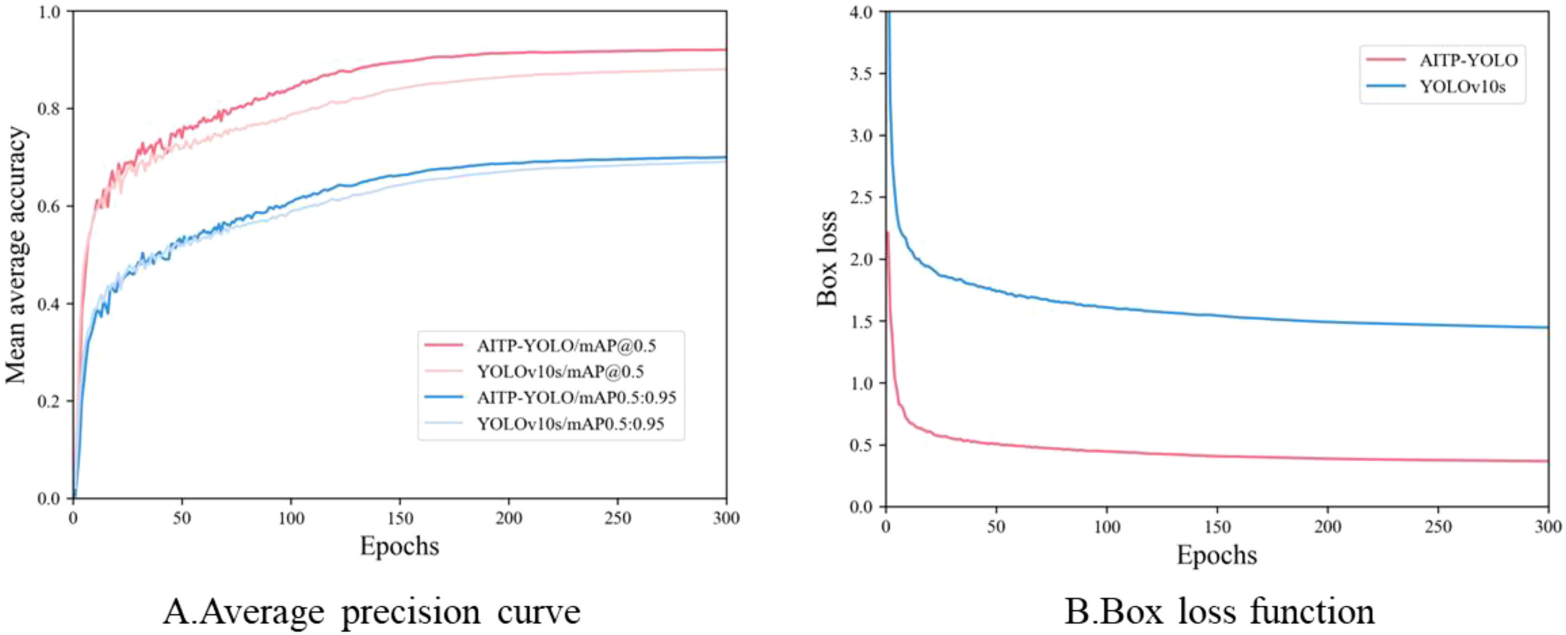
Training curves before and after model improvement. **(A)** the average precision curve, **(B)** the box loss function.

In order to verify the effectiveness of the model improvement, the average accuracy and the loss function of the model before and after the improvement are compared and analyzed. As shown in **Figure 10A**, AITP-YOLO performs significantly better than the baseline model YOLOv10s at the beginning of the training period, and its average accuracy improves faster and the accuracy curve is smoother, showing higher stability and robustness. As shown in **Figure 10B**, replacing Shape-IoU as the loss function accelerates the convergence of the model, the loss value continues to decrease, and the overlap between the predicted bounding box and the real bounding box increases significantly, thus effectively improving the overall performance of the model.

**Figure 11:**
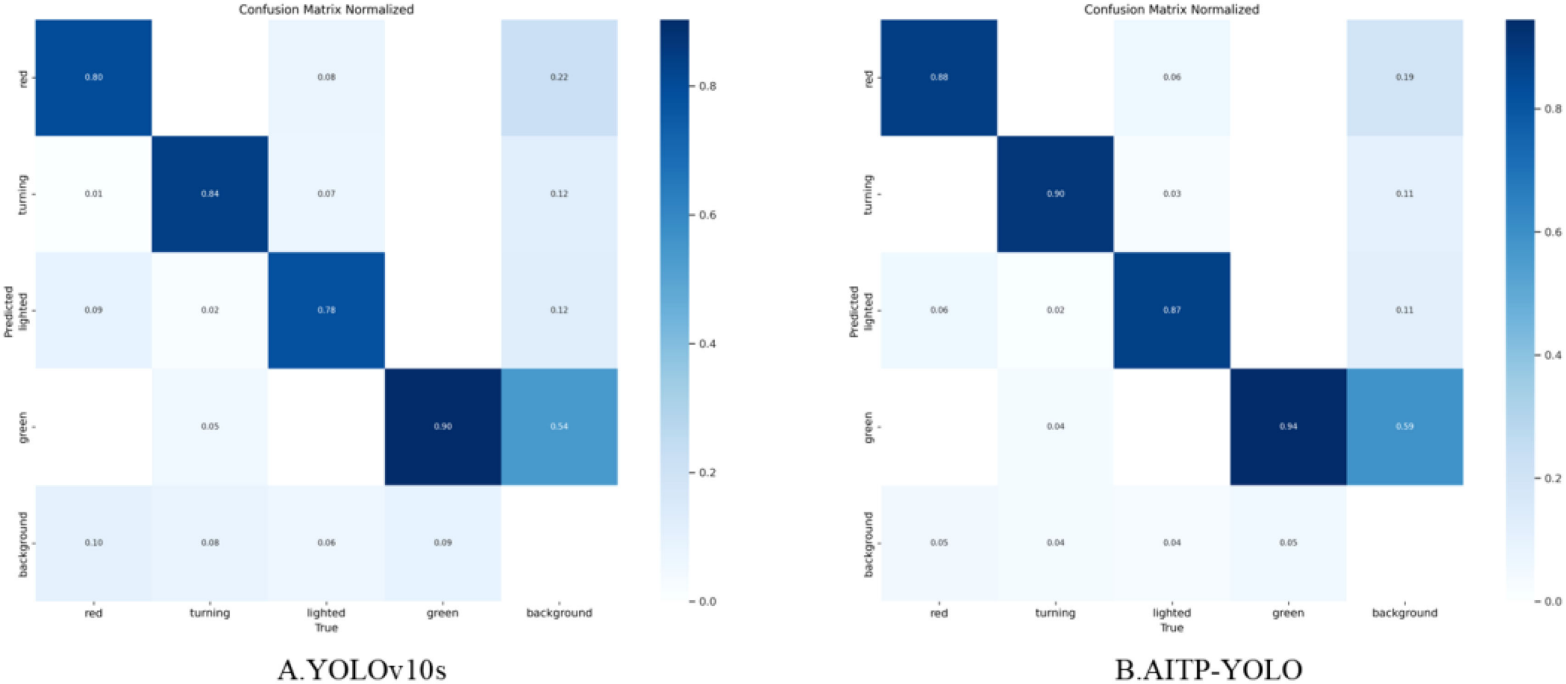
Improved pre- and post-model confusion matrices **(A)** YOLOv10s and **(B)** AITP-YOLO. The matrix was used to compare the performance of the two tomatoes in detecting ripeness (green, turning, lighted, red, and background categories), with darker colors representing higher prediction probabilities.

To further validate the performance of the AITP-YOLO model, a comparative analysis of the confusion matrices of the models before and during the enhancement is conducted, as illustrated in **Figure 11**. Analyzing the confusion matrices of YOLOv10s and AITP-YOLO reveals disparities in category recognition accuracy and misclassification rates. AITP-YOLO consistently enhances recognition accuracy across all four maturity groups as compared to YOLOv10s, which exhibits a comparatively elevated misclassification rate in each category, particularly in erroneously categorizing samples as belonging to the background category. This indicates that the final model has successfully minimized misclassification among categories through optimization, hence enhancing its capacity to differentiate between categories and that the optimization approaches have significantly improved its classification performance.

The results in **Table 7** indicate a considerable enhancement in the detection of tomato maturity at each stage, both before and following the improvement. In comparison to the baseline model YOLOv10s, the enhanced AITP-YOLO model improved recognition accuracy for the four ripening stages of tomatoes by 3.5%, 5.4%, 4.5%, and 4.7%, respectively, while reducing the model’s weight by 7.64%. This resulted in increases of 5.8, 7.3, 4.6, and 9 percentage points in accuracy, recall, and detection precision mAP@0.5 and mAP@0.5:0.95, respectively, thereby providing robust technical support for tomato ripeness.

**Table 7:** Comparison of results before and after the model is improved.

	Category	P/%	R/%	mAP@0.5/%	mAP@0.5:0.95/%
YOLOv10s	all	83.9	80.1	88	69.1
	green	86.8	85.5	91.7	68.2
	turning	82.6	81.6	88.1	72.1
	lighted	82.2	77.4	85.9	72.1
	red	83.9	76	86.4	69.1

	Category	P/%	R/%	mAP@0.5/%	mAP@0.5:0.95/%
AITP-YOLO	all	89.7	87.4	92.6	78.2
	green	90.9	91.4	95.2	76.5
	turning	91.4	87.8	93.5	81.1
	lighted	86.9	85.8	90.4	80.5
	red	89.7	84.8	91.1	74.5

### 4.6 Comparative experiments of different detection models

This study validates the efficacy of the AITP-YOLO model by comparing it with mainstream target detection models from the YOLO family and assessing their compatibility with the AITP model. This study conducted comprehensive comparisons of the AITP-YOLO model with other prominent convolutional neural network target detection models, including the two-stage target detection model Faster R-CNN (Ren et al., 2015) and the single-stage target detection algorithm SSD (Liu et al., 2016), to further validate the model’s efficacy. The findings are displayed in **Table 8**.

**Table 8:** Comparison experiments of different models.

model	P/%	R/%	mAP@0.5	mAP@0.5:0.95	Weight/MB	GFLOPs	FPS
Faster R-CNN	58.5	67	65	35.89	136.8	401.8	24.10
SSD	77.3	61.4	73.5	49.56	92.1	274.5	46.99
YOLOv5s	83.1	79.1	86.9	67.2	17.6	24	118.76
YOLOv8s	87	82.3	89.1	71.6	21.4	28.7	125
YOLOv10s	83.9	80.1	88	69.1	15.7	24.8	103.09
YOLOv11s	86	82.6	89.2	71.5	18.3	21.6	111.36
v8s-AITP	89.1	85.6	91.3	68	20.7	37.2	116.82
v10s-AITP	88.7	86.4	92.1	70	16.3	37.3	95.97
v11s-AITP	88.5	84.4	91.1	67.5	18.8	29.4	103.31
**AITP-YOLO**	**89.7**	**87.4**	**92.6**	**78.2**	**14.5**	**31.5**	**97.61**

The bold values represent the results of the optimal model AITP-YOLO in each comparison experiment.

The trials demonstrated that the AITP-YOLO model attains the greatest mAP@0.5 value, reaching 92.6%. The AITP model and the YOLO series of detection frameworks exhibit excellent interoperability, facilitating seamless integration with various backbone structures, and demonstrate substantial performance enhancements across the board. The Faster R-CNN and SSD models exhibit subpar performance in identifying small targets within intricate surroundings, with their detection efficacy markedly inferior to that of the YOLO series models. The results unequivocally illustrate the benefits and efficacy of the AITP-YOLO model in target identification tasks.

### 4.7 Model visualization results

**Figure 12:**
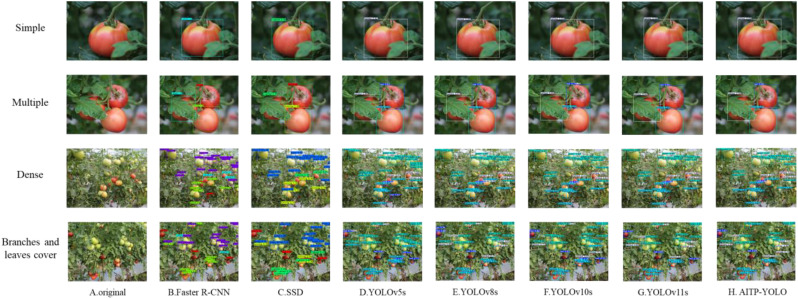
Comparison of visual inspection results of different models **(A)** original **(B)** Faster R-CNN **(C)** SSD **(D)** YOLOv5s **(E)** YOLOv8s **(F)** YOLOv10s **(G)** YOLOv11s **(H)** AITP-YOLO.

**Figure 12** illustrates the disparity in detection performance among the YOLOv5s, YOLOv8s, YOLOv10s, YOLOv11s, and AITP-YOLO models, primarily highlighting their detection capabilities in scenarios involving single fruits, multiple fruits, dense fruit clusters, and occlusions caused by branches and leaves. The Faster R-CNN and SSD models are susceptible to missed and incorrect detections in scenarios including dense fruits and occlusion by branches and leaves. The YOLOv5s model exhibits low overall identification accuracy, making it challenging to reliably identify tomato types. The YOLOv11s model struggles to reliably identify tomato fruits obscured by branches and leaves. The overall accuracies of YOLOv8s, YOLOv10s, and YOLOv11s are inferior to those of the AITP-YOLO model, which exhibits the best overall detection accuracy and is less prone to omissions or misdetections. Consequently, the AITP-YOLO model achieves both a lightweight design and an enhanced detection capability.

### 4.8 Edge device deployment

In the current advancement of agricultural intelligence, edge devices are important for achieving real-time data processing in the field due to their low power consumption and high adaptability. This study evaluates the suitability of the final model AITP-YOLO for deployment on edge devices by utilizing the Jetson Orin Nano Super (Gomes et al., 2022), a compact edge device, to achieve high frame rate operation of the AITP-YOLO model using a self-constructed tomato ripening dataset, thereby offering robust support for practical agricultural applications.

**Figure 13:**
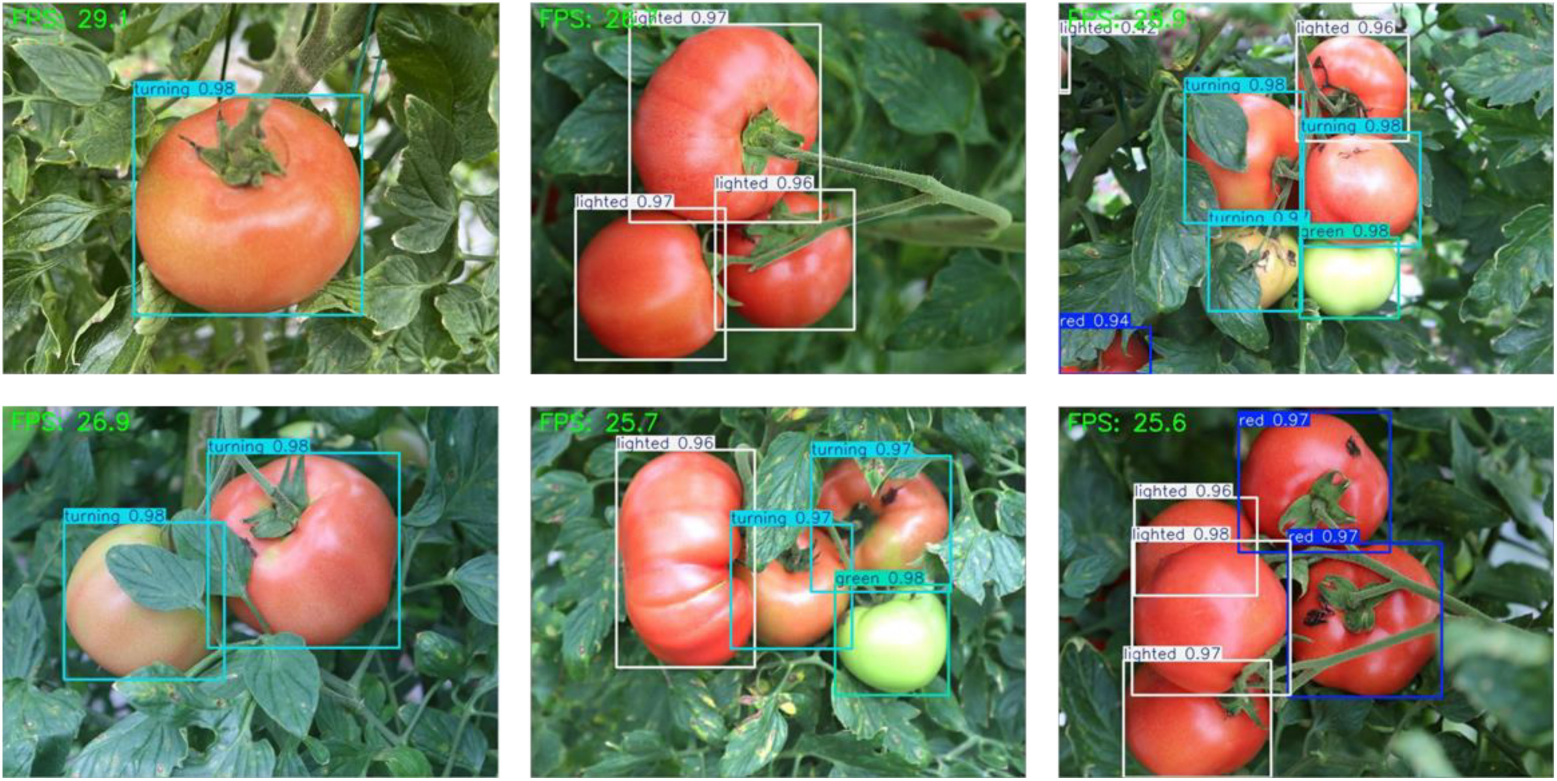
Jetson Orin Nano Super in action.

During the deployment procedure, the AITP-YOLO model files, formatted in PyTorch and trained on the self-assembled tomato ripening dataset, are initially sent to the edge device. Thereafter, the model is transformed into TensorRT format with the conversion tool offered by the YOLO framework, which significantly enhances model inference speed. The FP16 mixed-precision quantization technique is subsequently employed to transform the model weight data type from FP32 to half-precision floating-point integers, thereby enhancing inference speed while maintaining precision loss within acceptable limits. Upon finalizing the model deployment, the device is utilized to identify the target within the video stream. **Figure 13** illustrates the demonstration effect of the actual test.

The AITP-YOLO model operates consistently at an average frame rate of 27.15 FPS on the Jetson Orin Nano Super, as evidenced by the aforementioned data. This frame rate excels in edge device applications and satisfies real-time needs.

This study conclusively demonstrates the appropriateness of the AITP-YOLO paradigm for implementation on edge devices by deploying it on the Jetson Orin Nano Super. Its consistent frame rate, robust stability, and effective adaptability to compact edge devices offer a dependable technical solution for practical applications like smart agricultural harvesting, which holds significant potential for widespread use.

## 5 Conclusion and prospect

In recent years, with the advancement of agricultural modernization, tomato ripeness detection has gradually become a research hotspot, and many researchers are committed to developing target detection algorithms for tomato ripeness detection. The YOLO family of models is rapidly utilized in this domain due to its notable advantages of high real-time performance, balanced accuracy, and adaptability. Nonetheless, an examination of previous studies indicates that there remains potential for enhancement regarding accuracy, weight efficiency, and detection velocity. Some models exhibit issues related to substantial capacity and challenging deployment, while others attain lightweight or specialized features, their detection accuracy remains inadequate.

This work presents a lightweight AITP-YOLO model, enhanced from the YOLOv10s model. This model incorporates several multi-strategy enhancements: first, it integrates deep and shallow features by introducing a minor target detection layer to improve small target detection; second, it employs a multi-scale fusion strategy to address complex backgrounds; third, it substitutes the loss function with Shape-IoU to refine bounding box regression; and finally, it utilizes the Network Slimming pruning method for channel pruning and fine-tuning, thereby compressing the model and enhancing detection accuracy. Ablation studies on the proprietary Tomato dataset substantiate each enhancement. In comparison to the prevalent YOLO models, including YOLOv8s, YOLOv10s, and YOLOv11s, the mAP@0.5 of AITP-YOLO exhibits enhancements of 2.2%, 4.1%, and 1.9%, respectively. Consequently, AITP-YOLO demonstrates enhanced accuracy in detecting tomato ripeness amidst intricate backgrounds, achieving real-time detection at 97.61 FPS and 27.15 FPS on the Jetson Orin Nano Super edge device, making it suitable for deployment on mobile terminals or edge segments, thereby offering an efficient technological solution for intelligent fruit harvesting in agriculture. Effective technological solution for intelligent fruit harvesting in agriculture.

Despite the AITP-YOLO model’s commendable performance, opportunities for enhancement remain. Due to the potential for background confusion to result in misdetection and omission, the feature fusion module will be tuned to enhance accuracy, and more sophisticated model compression techniques will be investigated. The model is currently validated solely through computer simulation and has not been used in practice. Future trials will be conducted in tomato cultivation fields using picking robots to assess the robots’ practicality and reliability. The findings of this research align with existing peer studies, focus on enhancing the efficacy of tomato ripeness detection, and provide superior accuracy, reduced weight, and increased detection speed in integrated performance.

## Data Availability

The raw data supporting the conclusions of this article will be made available by the authors, without undue reservation.
